# Incidence and associated factors for hypotension after spinal anesthesia during cesarean section at Gandhi Memorial Hospital Addis Ababa, Ethiopia

**DOI:** 10.1371/journal.pone.0236755

**Published:** 2020-08-13

**Authors:** Tewoderos Shitemaw, Bedru Jemal, Temesgen Mamo, Luelayehu Akalu

**Affiliations:** 1 Anesthesia Department, Menelik II health and medical science college, Kotebe Metropolitan University, Addis Ababa, Ethiopia; 2 Department of Anesthesiology, College of Health Sciences and medicine, Dilla University, Dilla, Ethiopia; 3 Department of Anesthesiology, College of Health Sciences and medicine, Wachamo University, Wachamo, Ethiopia; 4 Department of Anesthesiology, College of Health Sciences and medicine, Addis Ababa University, Addis Ababa, Ethiopia; Federal University of Sergipe, BRAZIL

## Abstract

**Background:**

Spinal anesthesia is a type of regional anesthesia that has been practicing for obstetric anesthesia since the beginning of the 20th century. Despite the simplicity and lower maternal mortality risk, compared to general anesthesia, spinal anesthesia is linked to different adverse effects, of which hypotension is the most common complication. The main aim of this study was to determine the incidence and associated factors of hypotension after spinal anesthesia during cesarean section.

**Method:**

Institution-based cross-sectional study was conducted with 410 clients. The study was conducted on cesarean section under spinal anesthesia from 5^th^ January to 30^th^ April 2019, at the Gandhi Memorial Hospital, Addis Ababa, Ethiopia. Both bivariable and multivariable logistic regression analysis were done on the associated factors. The level of statistical significance was represented at p<0.05.

**Results:**

The incidence of hypotension among mothers who underwent a cesarean section after spinal anesthesia was 64%. Newborn weight ≥4kg (AOR = 5.373; 95%CI: (1.627–17.740)) showed an increase risk of association with hypotension. A baseline systolic blood pressure < 120mmHg (AOR = 6.293; (95%CI: 2.999–13.204)) was found to be associated with increased risk of hypotension. Sensory block height >T6 AOR = 2.230; 95%CI: (1.329–3.741), the time interval between spinal induction and skin incision > 6minutes AOR = 1.803; 95%CI: (1.044–3.114) and anesthetist experience AOR = 5.033(95%CI: 2.144–11.818) were also associated with hypotension.

**Conclusion:**

The identified risk factors for hypotension, after spinal anesthesia are sensory height block, weight of the baby, the time interval between spinal induction and skin incision, baseline systolic blood pressure, and anesthetist experience.

## Introduction

In Ethiopia, there is a high increase in practice of spinal anesthesia [1] Spinal anesthesia is preferred by both mothers and health professionals because of its unique advantages. Spinal anesthesia is a type of regional anesthesia that has been practiced for obstetric anesthesia since the beginning of the 20^th^ century. Nowadays regional anesthesia has become the most preferred method for cesarean section because of its simplicity and rapid onset along with maternal comfort and safety [2]. In Ethiopia, the prevalence of cesarean section was 32.5% and 68.2% CS was done by using spinal anesthesia [1].

Despite of the simplicity and lower maternal mortality risk compared to general anesthesia, spinal anesthesia has been linked to different adverse effects, out of which hypotension is most common complication [1]. The incidence of hypotension is 25–75% in general population and even higher in parturient who are undergoing cesarean section due to physiological change of pregnancy that causes compression of inferior vena cava by hypertrophic uterus and also the development of collateral venous plexus circulation in epidural space. This physiological change results in increased pressure of the cerebrospinal fluid (CSF) in the lumbosacral area, which results in cephalad spread of local anesthetics [3–5].

There has been little research examined the incidence and associated factors of hypotension after spinal anesthesia in developing countries. And out of the available studies, pregnant women have been excluded from most of the previous investigations. Therefore, identifying these factors associated with hypotension is paramount in an area where resources have been scarce for monitoring vital organs and also for guiding clinical decisions in mothers who are at risk of hypotension. The main purpose of this study is to assess the incidence of hypotension and also to determine maternal, fetal, and anesthesia-related factors associated with hypotension after spinal anesthesia in parturient undergoing cesarean delivery.

## Materials and methods

This study was an institutional-based cross-sectional study conducted at Gandhi memorial hospital found in Addis Ababa from 5^th^ January to 30^th^ April, 2019. The ethical clearance was obtained from Ethical Review Committee Addis Ababa University, Ethiopia, Africa. A formal letter from Addis Ababa University was submitted to Gandhi memorial hospital. All clients who fulfilled the inclusion criteria were presented with the objectives and rationale for the study and were informed of their right to stop the interview at any time if they wish, without giving any reason. The interviewers discussed the issue of confidentiality and obtain verbal consent before the actual interview was launched.

The methodology in this study is based on the international guidelines for observational studies according to Strengthening the Reporting of Observational Studies in Epidemiology (STROBE) 2010 statement (Supplementary-STROBE checklist) (S1 File).

All patients met the criteria of the American Society of Anesthesiologists (ASA) Physical Status II was included. The exclusion criteria were as follows: failed spinal and those patients who received a combination of spinal and general anesthesia.

The Data were collected from selected study participants using a pretested questionnaire in order to maintain the quality and consistency of data. The questionnaire mainly addressed socio-demographic variables (age, BMI, ASA status, and baby weight), maternal variables(gravidity, indication for C/History of hypertension preoperative Hgb and heart rate), anesthetic and surgical variables (LA dose, preload, intraoperative fluids, time interval b/n spinal induction and skin incision, type of surgery (elective and emergency), estimated blood loss, surgeon and anesthetist experience, a dose of uterotonic drug used, any pre and intraoperative drugs used adjuvants used and sensory block height).

The perioperative management was according to the established protocol of the institution: On arrival to operation theatre, all patients have an intravenous line placed and premedicated with IV cimetidine, metoclopramide, and ondasetrone 30 minutes before induction of anesthesia, for prevention of aspiration. Noninvasive monitoring consisted of non-invasive blood pressure (NIBP), electrocardiograph and pulse oximetry was used intraoperetively. The mean value of the first three consecutive measurements before commencing SA was defined as baseline blood pressure. Under strict aseptic precaution, SA was performed in sitting position by injection of a local anesthetic with or without opioid into the subarachnoid space. Immediately after the intrathecal injection, the patient was kept in supine left lateral tilt position. Sensory block height was measured by loss of cold sensation to alcohol swabs 10 min after induction of SA. After ensuring the appropriate level of blockade, surgery was started.

The sample size was determined by using single population proportion method for the first objective, which is the incidence of hypotension after spinal anesthesia. Since there is similar study conducted in study setting P = 0.5(prevalence of hypotension 50%) was used for the calculation to get the maximum sample size, 95% level of significance, 5% margin of error, and adding a 10% non-response rate yielding 422 study participants. A study participant was selected using a systematic random sampling technique with skip intervals from the daily operation scheduled for patients to undergo a cesarean section.

The collected data were checked for completeness and consistency. The data was entered in to Epidata version 3.1 and exported to SPSS 25 for analysis. Descriptive statistics was used to summarize tables and figures and statistical summary measures were used for presentation. Outlier of the data was checked using standardized residual tests and multi-collinearity for continuous data was checked by VIF and tolerance. Linearity of the continuous variables with respect to the logit of the dependent variable was assessed via the Box-Tidwell procedure and all continuous independent variables were found to be linearly related to the logit of the dependent variable. Bivariate logistic regression analysis has been performed to see significance of association between dependent and independent variables. All explanatory variables which had association in bivariate analysis at p-value less than or equal to 0.25 were entered into multivariable logistic regression model by back ward elimination method. P-value < 0.05 was considered to be risk factor for hypotension after spinal anesthesia during cesarean section in this study. The results of significant variables were presented as frequency table, crude and adjusted OR with 95% CI. The goodness model was tested by Hosmer Lemeshow test and the model was best fit with P-Value 0.615.

## Result

### Socio-demographic characteristics of respondents

During the study period, 410 out of 422 clients were available for the final analysis with a response rate of 97%. The mean (SD) age of the mothers was 27.6 (4.23) year. Regarding the maternal height, most of them are at height range from 160-169cm (63.7%), and around a quarter of them are at a height from 150-159cm Table 1.

**Table 1:** Socio-demographic characteristics of pregnant mothers who undergone C/S under spinal anesthesia.

Variables	Category	Number	Percent
Age(year)	15–25	120	29.3
	26–35	250	61
	>35	40	9.8
Height(cm)	150–159	97	23.7
	160–169	261	63.7
	170–179	52	12.7
BMI(kg/m^2^)	18.5–24.9	186	45.4
	25–29.9	143	34.9
	≥30	81	19.8
ASA	class 2	410	100
Baby weight(kg)	≤2.4	36	8.8
	2.5–3.9	314	76.6
	≥4	60	14.6

**Fig 1:**
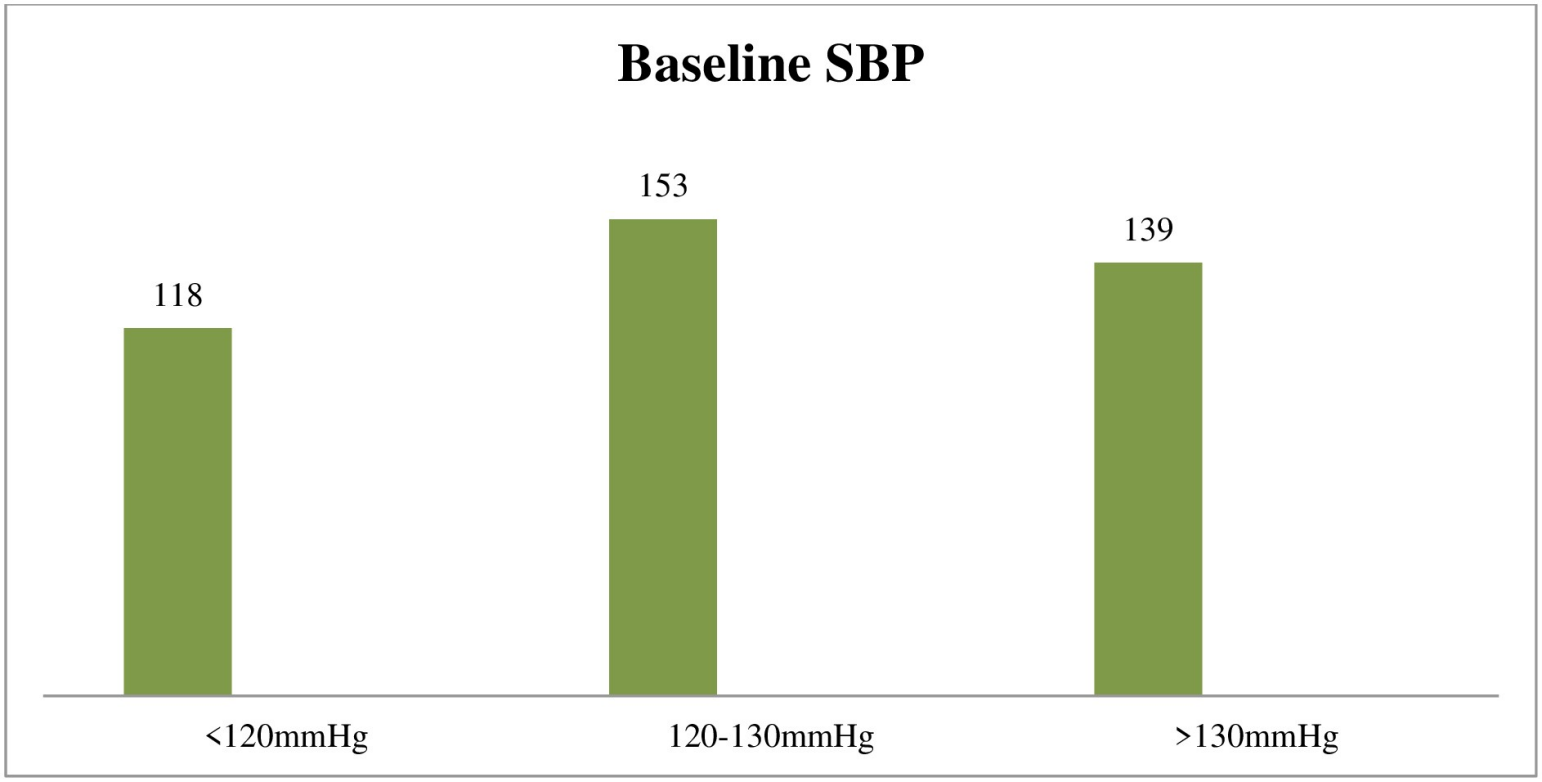
Baseline SBP of clients who underwent cesarean section under spinal anesthesia.

From all participants in study 153(37.3%) had baseline blood pressure of 120-130mmHg which is relatively high (Fig 1).

### Obstetric characteristics, medical and preoperative related history

**Fig 2:**
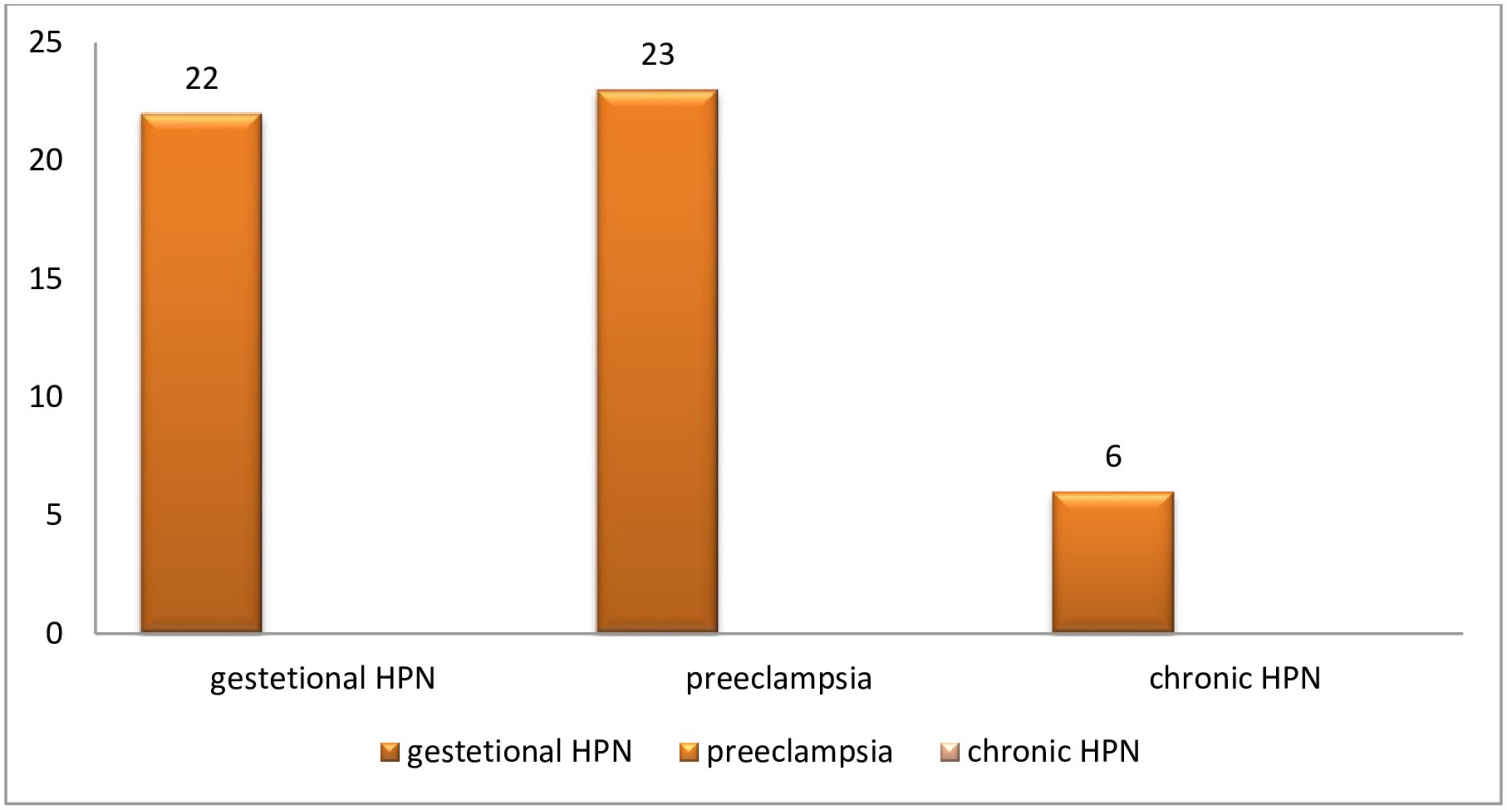
Types of hypertension.

From all study participants, 263 (64.1%) had a preoperative hemoglobin level of <11mg/dl and the rest were above 11g/dl. The most common indication for cesarean section was malpresentation of fetus 130(31.7%) followed by non-reassuring fetal status 121(29.5%). Most of the cesarean sections have been done by year three resident 195(47.6%) and year four resident 123(30%) Table 2. Of all clients who came for C/S, 51(12%) had a hypertensive disorder. Gestational hypertension and preeclampsia account for 23(5.6%) and 22(5.4%) of types of hypertensive disorder respectively (Fig 2).

**Table 2:** Obstetric characteristics of pregnant mothers who undergone C/S under spinal anesthesia.

variable	Category	Number	Percent
Preoperative hemoglobin	Above 11g/dl	147	35.9
	Below 11g/dl	263	64.1
gravidity	One	104	25.4
	Two	145	35.4
	Three	108	26.3
	Four	36	8.8
	Five	17	4.1
Indication for C/S	Malpresentation	130	31.7
	Non-reassuring fetal status	121	29.5
	Previous scar	79	19.3
	Previous scar and labour	36	8.8
	Other indication	44	10.7
Amount of crystalloids preloaded	< 500	227	55.4
	>500	183	44.6
Experience of surgeon	Resident two	77	18.8
	Resident three	195	47.6
	Resident four	123	30
	Senior	15	3.7
hypertension	No	359	87.5
	Yes	51	12.5

### Anesthesia characteristics and drug

The majority of the anesthetists administered 0.5% of 12.5mg bupivacaine. Fentanyl 25 mcg was added as an adjuvant to bupivacaine for 48(11.7%) mothers undergone a cesarean section. The sensory block height below T6 was achieved on 49.7% of the clients Table 3.

**Table 3:** Characteristics of spinal anesthesia and drug used for prevention hypotension.

Variables	Category	Number	Percent
Site of spinal puncture	L2-L3	15	3.7
	L3-L4	385	93.9
	L4-L5	10	2.4
Spinal needle size	20G	17	4.1
	21G	38	9.3
	22G	162	39.5
	23G	170	41.5
	25G	23	5.6
Dose of LA with adjuvants(fentanyl)	10mg	31	7.6
	12.5mg	296	72.2
	15mg	35	8.5
	10mg &25mcg	48	11.7
Sensory height block	>T6	202	49.3
	≤T6	208	50.7
Adjuvants used with LA	No	362	88.3
	Yes	48	11.7
Any prophylactic vasoactive drug given	Yes	9	2.2
	No	401	97.8
Dose of adjuvants used with LA	fentanyl 25mcg	48	11.7
Experience of anesthetist	One year	74	18
	Two years	86	21
	Three years	51	12.4
	Four years	43	10.5
	Above four years	93	22.7
	Bsc students	63	15.4

### Intraoperative variable

Majority of the study participants 394 (77.8%) have received oxytocin for uterine contraction and the rest have been received both oxytocin and Ergometrine together. Regarding the intraoperative fluid majority of the study, participants had taken greater than 1500ml of fluid (52.2%) followed by 1000-1500ml (28.5%) of 0.9% normal saline Table 4.

**Table 4:** Intraoperative variable.

Variables	Category	Number	Percent
Types of uterotonic drugs used	Oxytocin & Ergometrine	16	4
	Oxytocin	394	96
Amount of crystalloid intraoperative	<1000	79	19.3
	1000–1500	117	28.5
	>1500	214	52.2
Dose of uterotonic drugs used	Ergometrine 0.25mg & oxytocin 20IU	16	3.9
	Oxytocin 20IU	319	77.8
	Oxytocin 30IU	18	4.4
	Oxytocin 10IU	57	13.9
Estimated blood loss	500–999	381	92.9
	≥1000	29	7.1
Time interval b/n spinal induction and skin incision	5	134	32.7
	>6	276	67.3

### Incidence of spinal anesthesia induced hypotension

**Fig 3:**
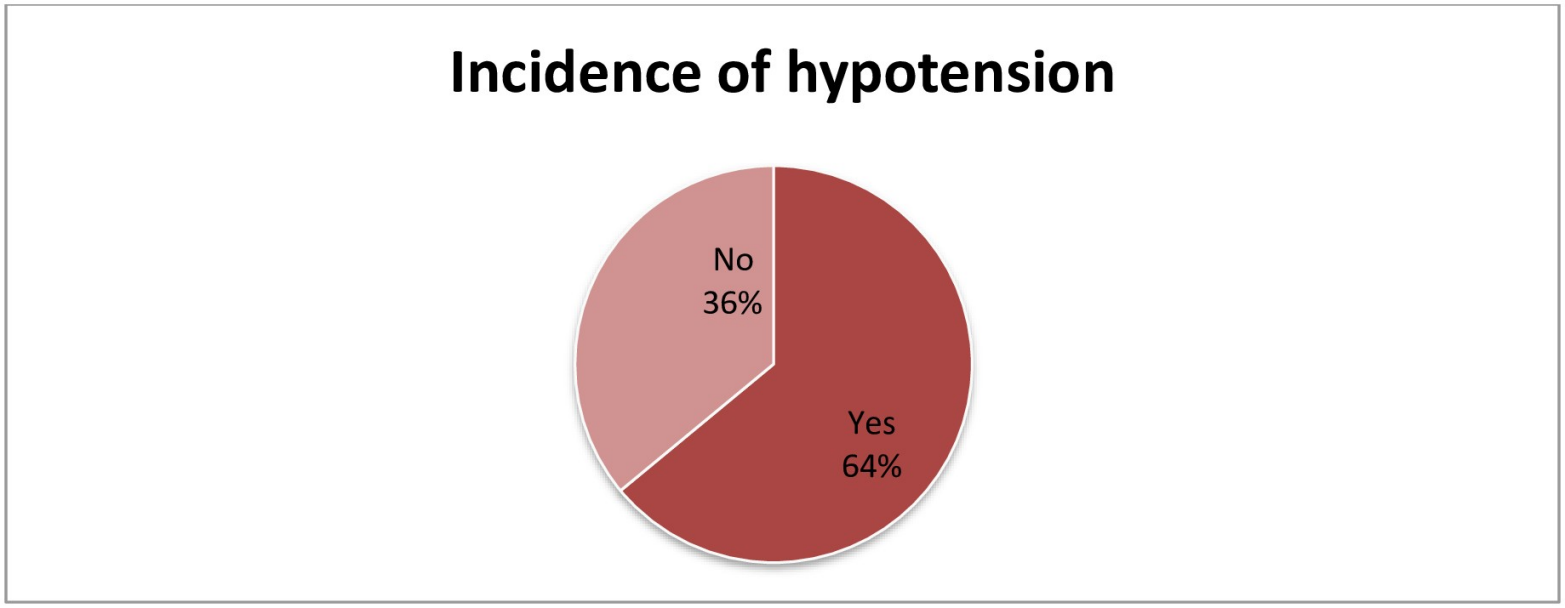
Incidence of hypotension among women’s who undergone cesarean section under spinal anesthesia.

From the total pregnant mothers who underwent a cesarean section under spinal anesthesia, the incidence of hypotension was present in 263 (64%) of the patients (Fig 3).

### Socio-demographic, preoperative and intraoperative factors associated with hypotension during cesarean section after spinal anesthesia

Among all factors included in multivariate analysis preload of crystalloid, estimated blood loss, preoperative hemoglobin, gravidity, indication for cesarean section, the experience of surgeon, body mass index, maternal age, the dose of local anesthetics and adjuvants used, type of uterotonic drug and time interval between skin incision and delivery were not significantly associated factors for hypotension S1–S3 Tables.

### Factors associated with hypotension after spinal anesthesia for cesarean section

The finding from multiple logistic regressions revealed that newborn weight, baseline SBP, sensory block height, time interval between spinal induction and skin incision and experience of anesthetists were found to be significantly associated with hypotension.

Newborn weight of 2.5–3.9kg (AOR = 3.414; 95% CI: (1.392–8.375)), newborn weight ≥4kg (AOR, 5.3(95% CI: 1.6–17.7), baseline systolic blood pressure less than 120mmHg (AOR, 6.293(95% CI: 2.99–13.2), sensory block height > T6 (AOR = 2.230; 95%CI: (1.329–3.741)), time interval between spinal induction and skin incision> 6 minute (AOR = 1.803;95%CI: (1.044–3.114)) and experience of anesthetist < one year (AOR = 5.033(95%CI:2.144–11.818) had shown association with hypotension Table 5.

**Table 5:** Multivariate analysis showing factors associated hypotension among pregnant mothers who undergone C/S under spinal anesthesia.

Variables	Category	Had hypotension		COR 95% CI	AOR 95% CI	p-value
		No	Yes			
Baby weight	≤2.4kg	23	13	1	1	
	2.5–3.9kg	111	203	3.236(1.578–6.637)	3.414(1.392–8.375)**	0.007
	≥4kg	13	47	6.396(2.558–15.993)	5.373(1.627–17.740)**	0.006
Baseline SBP	>130mmHg	72	67	1	1	
	120-130mmHg	53	103	2.214(1.378–3.557)	3.8(2.079–6.945)***	0.0001
	<120mmHg	25	93	3.998(2.300–6.949)	6.293(2.999–13.204)***	0.0001
Sensory height block	<T6	100	102	1	1	
	>T6	47	161	3.358(2.193–5.143)	2.230(1.329–3.741)**	0.002
interval b/n anesthesia and skin incision	≤5 minutes	57	77	1	1	
	>6 minutes	90	186	1.530(1.000–2.340)	1.803(1.044–3.114)**	0.035
Anesthetist experience	Above five year	51	42	1	1	
	Four years	22	21	1.59(0.562–2.391)	0.965(0.411–2.265)	0.934
	Three years	16	35	2.656(1.295–5.450)	3.059(1.263–7.409)**	0.013
	Two years	23	63	3.326(1.774–6.235)	5.094(2.326–11.158)***	0.0001
	One year	20	54	3.279(1.702–6.316)	5.033(2.144–11.818)***	0.0001
	BSc students	15	48	3.886(1.912–7.897)	5.062(2.132–12.020)***	0.0001

Statistically significant ** P < 0.05, ***P<0.001, AOR adjusted odds ratio.

## Discussion

In our study, the overall incidence of hypotension among mothers undergone a cesarean section with spinal anesthesia is 64%. Previous studies have been examined the incidence of hypotension after spinal anesthesia for C/S revealing different results. A prior study in Ethiopia was observed hypotension incidence of 36% after spinal anesthesia. Other studies in different parts of the world; have reported 65.1%, 76.7%, 80%, and 56.5% incidence of hypotension. The possible reason for the different incidence might be due to different methods of measurement, the operational definition of hypotension, and clinical setup [1, 6–10].

Our study showed that baby weight, baseline systolic blood pressure, sensory block height, the time interval between spinal induction and skin incision, and experience of anesthetists were factors associated with hypotension.

Mothers with a newborn weight of ≥4kg were five times more at risk of developing hypotension than a newborn weight of ≤2.4kg weight (AOR, 5.3(95% CI: 1.6–17.7). This findings agrees with the previous studies finding in which the weight of the newborn was identified as a risk factor for developing hypotension when a newborn’s weight is greater than 3900 grams [10, 11]. The reason might be as the baby’s weight increases the risk of inferior vena cava and major artery compression by gravid uterus increase. This compression will result in a decreased venous return which leads to a reduction in preload and predisposes to hypotension.

Another factor associated with hypotension in this study was baseline systolic blood pressure. A baseline systolic blood pressure of <120mmHg with (AOR, 6.293(95% CI: 2.99–13.2) and 120-130mmHg with (AOR, 3.800(95% CI: 2–6.9) had six and three folds of developing hypotension compared to SBP ≥130mmHg respectively. This finding is consistent with study done in Bangkok that stated the risk of hypotension in patients with baseline SBP less than 130mmHg increased [9] and according to Randall L and colleagues baseline systolic blood pressure less than 120mmHg has a 2-fold increase in the odds of developing hypotension which supports our finding [12]. The reason behind this will be a higher baseline blood pressure has high safety of margin. The other possible reason might include, those clients with low baseline systolic blood pressure may have low baseline systemic vascular resistance that increases the risk of hypotension.

The sensory height block higher than T6 (AOR 2.23(95%CI: 1.3–3.7) resulted two times more risk of developing hypotension. This finding agrees with previous findings. Such as, the finding in Chulalongkorn university, Bangkok which shows the sensory block height greater than T5 was associated with a two folds increase in the risk of hypotension [8]. The possible explanation related to sensory block height is due to blockade of sympathetic nerve outflow. Furthermore, higher sensory levels of analgesia correlated with a relatively greater reduction in systolic blood pressure [6]. The physiological explanation is that the higher the level of sensory blockade, the more autonomic blockade resulting a loss of vasomotor tone and cause hypotension [12, 13].

The risk of hypotension has a strong association with the experience of an anesthetist, our study shows the risk of hypotension was increased as the experience of anesthetist decrease compared to those who have more experience. The previous finding in Ethiopia strongly supports our findings [1].

The other finding which was associated with hypotension is the time interval between spinal induction and skin incision. The longer the time interval is between spinal induction and the skin incision, it is associated with an increased risk of developing hypotension. The possible explanation might be as the time gap increase between spinal induction and skin incision, the time of pregnant mothers being in a supine position without delivering the baby increase. This increase in both the aortocaval compression time and the sympathetic blockade by spinal anesthesia, exaggerating the risk of hypotension [14, 15]. Our study was limited in that we did not measure the speed of injection during spinal anesthesia and hemodynamic effect of spinal fentanyl because there is shortage of strong analgesics in the country and due to this adjuvant are not used consistently by anesthetists. Hence only around 10% of the data recorded adjuvant use which might have an impact on the outcome of the study.

In conclusion, the incidence of hypotension is 64% in this study, higher than previous studies in different countries. We found newborn weight, baseline SBP, sensory height, the experience of anesthetist, and time interval between spinal induction and skin incision as an independent risk factor for hypotension. We recommend Clinicians, to recognize these risk factors and be vigilant to manage the occurrence of hypotension. In addition, it’s our recommendation for longitudinal studies to recognize the long-term effect of spinal induced hypotension.

## Supporting information

S1 File STROBE checklist. (DOCX)

S1 Table Bivariate analysis showing factors associated with hypotension among pregnant mothers who undergone C/S after spinal anesthesia. (DOCX)

S2 Table Preoperative and maternal related factors associated with spinal anesthesia induced hypotension for cesarean section under spinal anesthesia. (DOCX)

S3 Table Anesthetic and surgical factors associated with hypotension among pregnant mothers who undergone cesarean section under spinal anesthesia. (DOCX)

## Abbreviations

AOR: Adjusted Odds Ratio
ASA: American Society of Anesthesiology
BMI: Body Mass Index
BSc: Bachelor of Science
C/S: Cesarean Section
CI: Confidence Interval
COR: Crude Odd Ratio
CSF: Cerebrospinal fluid
GA: General Anesthesia
L: Lumbar
LA: Local Anesthetic
OR: Odds Ratio
OT: Operating theatre
SA: Spinal Anesthesia
SBP: Systolic Blood Pressure
SIH: Spinal induced hypotension
SPSS: Statistical Packages for Social Science
