# Supplementary material for: Incidence and associated factors for hypotension after spinal anesthesia during cesarean section at Gandhi Memorial Hospital Addis Ababa, Ethiopia
Source: PLoS One. 2020 Aug 13;15(8):e0236755. doi: 10.1371/journal.pone.0236755 (PMC7425909; doi:10.1371/journal.pone.0236755)
Supplement: S1 Table — (DOCX) [file pone.0236755.s002.docx]

S1 Table. Bivariate analysis showing factors associated with hypotension among pregnant mothers who undergone C/S after spinal anesthesia.

| Variables | **Category** | **Had hypotension** | | **COR 95%CI** | **p-value** |
| --- | --- | --- | --- | --- | --- |
|  |  | **No** | **Yes** |  |  |
| Age(year) | 15-25 | 41 | 79 | 1 |  |
|  | 26-35 | 101 | 149 | **0.766(0.486-1.206)*** | 0.249 |
|  | >35 | 5 | 35 | **3.633(1.323-9.976)*** | 0.012 |
| BMI(kg/m^2^) | 18.5-24.9 | 76 | 110 | 1 |  |
|  | 25-29.9 | 51 | 92 | 1.246(0.79-1.955) | 0.338 |
|  | >30 | 20 | 61 | **2.107(1.176-3.777)*** | 0.012 |
| Height(cm) | 150-159 | 36 | 61 | 1.243(0.625-2.471) | 0.536 |
|  | 160-169 | 89 | 172 | 1.417(0.773-2.600) | 0.260 |
|  | 170-179 | 22 | 30 | 1 |  |
| Baby weight (kg) | <2.4 | 23 | 13 | 1 |  |
|  | 2.5-3.9 | 111 | 203 | **3.236(1.578-6.637)*** | 0.001 |
|  | >=4 | 13 | 47 | **6.396(2.558-15.993)** |  |
