# Supplementary material for: Incidence and associated factors for hypotension after spinal anesthesia during cesarean section at Gandhi Memorial Hospital Addis Ababa, Ethiopia
Source: PLoS One. 2020 Aug 13;15(8):e0236755. doi: 10.1371/journal.pone.0236755 (PMC7425909; doi:10.1371/journal.pone.0236755)
Supplement: S2 Table — (DOCX) [file pone.0236755.s003.docx]

S2 Table. Preoperative and maternal related factors associated with spinal anesthesia induced hypotension for cesarean section under spinal anesthesia.

| Variables | Category | Had hypotension | | COR95%CI | P-value |
| --- | --- | --- | --- | --- | --- |
|  |  | No | Yes |  |  |
| Preoperative maternal hemoglobin | Above 11g/dl | 45 | 102 | 1 |  |
|  | Below 11g/dl | 102 | 161 | 0.696(0.453-1.070) | 0.099 |
| Base line SBP | >130mmHg | 72 | 67 | 1 |  |
|  | 120-130mmHg | 53 | 103 | **2.214(1.378-3.557)*** | 0.001 |
|  | <120mmHg | 25 | 93 | **3.998(2.300-6.949)*** | 0.000 |
| Base line heart rate | 60-80 | 25 | 48 | 1 |  |
|  | 81-100 | 91 | 143 | 0.818(0.472-1.419) | 0.475 |
|  | >100 | 31 | 72 | 1.210(0.637-2.297) | 0.561 |
| Gravidity | One | 74 | 30 | 1 |  |
|  | Two | 61 | 84 | **1.791(1.047-3.065)*** | 0.033 |
|  | Three | 36 | 72 | 1.233(0.688-2.210) | 0.481 |
|  | Four | 13 | 23 | 1.394(0.625-3.108) | 0.416 |
|  | Five | 7 | 10 | 1.727(0.601-4.959) | 0.310 |
| Indication for C/S | Malpresentation | 83 | 47 | 1 |  |
|  | Non-reassuring fetal status | 82 | 39 | 0.840(0.498-1.417) | 0.525 |
|  | Previous scar | 53 | 26 | 0.866(0.480-1.563) | 0.634 |
|  | Previous scar and labour | 17 | 19 | **1.974(0.936-4.161)*** | 0.074 |
|  | Other indication | 28 | 16 | 1.009(0.496-2.054) | 0.980 |
