# Supplementary material for: Incidence and associated factors for hypotension after spinal anesthesia during cesarean section at Gandhi Memorial Hospital Addis Ababa, Ethiopia
Source: PLoS One. 2020 Aug 13;15(8):e0236755. doi: 10.1371/journal.pone.0236755 (PMC7425909; doi:10.1371/journal.pone.0236755)
Supplement: S3 Table — (DOCX) [file pone.0236755.s004.docx]

S3 Table. Anesthetic and surgical factors associated with hypotension among pregnant mothers who undergone cesarean section under spinal anesthesia.

| Variables | Category | Had hypotension | | | COR 95% CI | P-value |
| --- | --- | --- | --- | --- | --- | --- |
|  |  | No | Yes | |  |  |
| Sensory height block | <T6 | 100 | 102 | 1 | |  |
|  | >T6 | 47 | 161 | **3.358(2.193-5.143)*** | | 0.000 |
| Experience of anesthetist | Above four years | 51 | 42 | 1 | |  |
|  | Four years | 22 | 21 | 1.59(0.562-2.391) | | 0.689 |
|  | Three years | 16 | 35 | **2.656(1.295-5.450)*** | | 0.008 |
|  | Two years | 23 | 63 | **3.326(1.774-6.235)*** | | 0.000 |
|  | One year | 20 | 54 | **3.279(1.702-6.316)*** | | 0.000 |
|  | BSc students | 15 | 48 | **3.886(1.912-7.897)*** | | 0.000 |
| Time interval b/n spinal induction and skin incision | ≤5 minutes | 57 | 77 | 1 | |  |
|  | >6 minutes | 90 | 186 | **1.530(1.000-2.340)*** | | 0.05 |
| Interval b/n incision and delivery | < 3 minutes | 72 | 128 | 1 | |  |
|  | >3 minutes | 75 | 135 | 0.98(0.66-1.479) | | 0.952 |
| Estimated blood loss | 500-1000 | 243 | 138 | 1 | |  |
|  | >1000 | 20 | 9 | 1.262(0.559-2.848) | | 0.575 |
| Crystalloids preloaded | >500ml | 70 | 113 | 1 | |  |
|  | <500ml | 77 | 150 | 1.207(0.805-1.810) | | 0.364 |
|  | Oxytocin 10IU | 19 | 38 | 1 | |  |
|  | Oxytocin 20IU | 117 | 202 | 0.863(0.476-1.567) | | 0.629 |
|  | Oxytocin 30IU | 6 | 12 | 1.00(0.325-3.077) | | 1.000 |
|  | Ergo 0.25mg and oxy 20IU | 5 | 11 | 1.100(0.334-3.623) | | 0.875 |
| Experience of surgeon | Year two Resident | 29 | 48 | 1.892(0.621-5.764) | | 0.262 |
|  | Year three Resident | 68 | 127 | **2.134(0.742-6.138)*** | | 0.159 |
|  | Year four Resident | 42 | 81 | **2.204(0.748-6.495)*** | | 0.152 |
|  | Senior | 8 | 7 | 1 | |  |
| Fentanyl | Yes | 19 | 29 | 0.835(0.45-1.54) | | 0.567 |
|  | No | 128 | 234 | 1 | |  |

### *P< 0.25 COR crude odds ratio.
